# Late complications of the Rastelli procedure - infective endocarditis and homograft stenosis: A case report

**DOI:** 10.2478/jccm-2026-0007

**Published:** 2026-01-30

**Authors:** Elena-Andreea Sava, Ilie-Marius Ciorba, Claudiu-Ionut Sântean, Andrei Manea, Nicoleta-Maria Crăciun-Ciorba

**Affiliations:** Department of Cardiology, Targu Mures Institute for Cardiovascular Diseases and Heart Transplantation, Targu Mures, Romania; Department of Internal Medicine I, George Emil Palade University of Medicine, Pharmacy, Science, and Technology of Targu Mures,Romania; Department of Gastroenterology, Emergency County Hospital Targu-Mures, Targu Mures, Romania; Doctoral School of Medicine and Pharmacy, George Emil Palade University of Medicine, Pharmacy, Science, and Technology of Targu Mures,Romania; Department of Radiology, George Emil Palade University of Medicine, Pharmacy, Science, and Technology of Targu Mures,Romania; Department of Internal Medicine II, George Emil Palade University of Medicine, Pharmacy, Science, and Technology of Targu Mures,Romania; Department of Internal Medicine I, Emergency County Hospital Targu-Mures, Targu Mures, Romania

**Keywords:** transposition of great arteries, Blalock Taussing shunt, Rastelli procedure, pulmonary hypertension, infective endocarditis, heart failure

## Abstract

**Introduction:**

Advances in surgical techniques have significantly improved the prognosis of patients with operated congenital heart malformations. However, late complications pose a challenge to therapeutic management. Although the Rastelli procedure has brought substantial benefits in the surgical correction of transposition of the great arteries in pediatric patients, it carries the burden of numerous complications into adulthood.

**Case presentation:**

We present the case of a 35-year-old man diagnosed at birth with D-transposition of the great arteries, atrial septal defect, ventricular septal defect and severe pulmonary stenosis. His medical history revealed two previous operations: a Blalock-Taussing shunt at the age of 4 months and a Rastelli procedure at the age of 3 years. The patient presented to the emergency room with fever and congestive heart failure symptoms. Subsequent investigations revealed two late complications of the Rastelli procedure: stenosis of the homograft connecting the pulmonary artery to the right ventricle and infective endocarditis.

**Conclusions:**

Although the clinical context may lead to the assumption that this is a case of congestive heart failure due to homograft stenosis, we must not overlook the possibility of overlapping infective endocarditis, which may also contribute to the development of heart failure.

## Introduction

D-transposition of the great arteries is a complex congenital heart defect, characterized by atrioventricular concordance (right atrium to right ventricle, left atrium to left ventricle), but ventriculoarterial discordance specifically, the aorta arises from the right ventricle and the pulmonary artery from the left ventricle. This form typically requires urgent surgical intervention within the first month of life, most often through an elective arterial switch operation, which includes coronary artery reimplantation (commonly known as the Jatene procedure). In certain cases, alternative surgical approaches may be indicated, such as the Rastelli procedure, used when D-transposition of the great arteries is associated with pulmonary stenosis and a ventricular septal defect, or the atrial switch procedures (Mustard or Senning operations) [1, 2].

Regarding the therapeutic management of transposition of the great arteries in patients with severe symptoms, it is preferable to perform a shunt procedure connecting a systemic artery (usually the subclavian artery) to the pulmonary artery until definitive correction. Thus, as a first therapeutic step, Alfred Blalock, Helen B. Taussing and Vivien Thomas developed a shunt that mimics a patent ductus arteriosus and connects the right subclavian artery to the right pulmonary artery to increase pulmonary blood flow [3]. Although the Blalock-Thomas-Taussing shunt was a temporary correction, it provided a chance of survival for patients with congenital heart disease. One of the definitive correction methods for D-transposition of the great arteries associated with pulmonary stenosis and ventricular septal defect is the Rastelli procedure. In this procedure, the ventricular septal defect is closed with a Gore-Tex patch, allowing blood flow from the left ventricle to the aorta, while the right ventricle is connected to the pulmonary artery via a Gore-Tex conduit or homograft [4].

As with any surgical procedure, the Rastelli procedure exposes patients to certain complications. These include extra-cardiac conduit stenosis, as well as complications from the tunnel patch between the left ventricle and the aorta. Such complications may include leakage, stenosis, obstruction, or aneurysm. Other complications include pulmonary artery trunk stenosis, infective endocarditis, biventricular dysfunction, and third-degree atrioventricular block, which may require pacemaker implantation.

One of the most common complications that occurs after the Rastelli procedure is stenosis of the conduit connecting the right ventricle to the pulmonary artery, which usually occurs on average 7.8 years after surgery [5]. This stenosis can have multiple causes, the most common being compression of the conduit by the sternum and chest wall, especially in patients with pectus excavatum, and degeneration of the conduit with calcium accumulation. The most common method of correction is surgical replacement of the conduit, but in patients with pectus excavatum, partial sternectomy may be necessary to reduce the compression exerted by the chest skeleton on the conduit [6]. Additionally, if degenerative conduit stenosis is associated with pectus excavatum, the Nuss procedure (a minimally invasive repair of the chest wall) can be performed alongside conduit replacement.[7]

Although infectious endocarditis is rare, it is a life-threatening complication if not diagnosed and treated properly after the Rastelli procedure. Potential causes include the presence of prosthetic materials, such as the conduit and patch, which can serve as sites for infection to develop. The most common pathogens are various species of streptococci and staphylococci, including Staphylococcus aureus. However, endocarditis with negative blood cultures can account for up to 20% of cases [8]. Diagnosing this pathology is often challenging. The first imaging method is transthoracic echocardiography. If there are no signs of endocarditis, the next steps in the diagnostic process include transesophageal echocardiography, cardiac MRI, and positron emission tomography with fludeoxyglucose. Treatment usually involves prolonged intravenous antibiotic therapy, and in some cases, surgery is necessary to address complications such as pseudoaneurysms and severe valve regurgitation.

## Case Presentation

We present the case of a 35-year-old Caucasian man who presented to the emergency department with fever, shortness of breath, tachypnea, weakness and fatigue. His medical history includes a birth diagnosis of D-transposition of the great arteries, atrial and ventricular septal defects and severe pulmonary stenosis. At the age of 4 months, he underwent a Blalock-Thomas-Taussing shunt (a surgical procedure in which the right subclavian artery is connected to the right pulmonary artery to increase the blood flow to the lungs). Subsequently, at the age of 3, he underwent corrective surgery for transposition of the great arteries using the Rastelli procedure (the ventricular septal defect was closed with a Gore-Tex patch connecting the left ventricle to the aorta, and a homograft was placed between the right ventricle and the pulmonary artery), the atrial septal defect was closed, and the Blalock shunt was removed. In addition, the patient has stage IIIB chronic kidney disease, is a chronic wearer of a urinary catheter, and has a history of multiple hospitalisations for sepsis of unknown origin, which were treated with empiric antibiotic therapy.

On admission, the patient was conscious with a Glasgow Coma Scale (GCS) score of 15, febrile (39°C), oxygen saturation of 75% on room air and 90% with a nonrebreather mask. Physical examination revealed a post-sternotomy scar, pectus excavatum, no skin pallor or rash, no adenopathy, distention of the jugular veins, absence of vesicular breath sounds bilaterally in the lower half of the lung fields, a grade IV/6 systolic murmur with panfocal radiation, blood pressure of 110/60 mmHg without positive inotropes, respiratory rate of 26 breaths/min, qSOFA score of 1 point and abdominal distension without pain on palpation. Laboratory tests revealed leukocytosis, neutrophilia, moderate anaemia, elevated inflammatory markers, a high NT-proBNP level and acute renal failure (Table 1). Urinalysis showed 500 leucocytes/μL and positive nitrites and arterial blood gas analysis showed mixed acidosis (metabolic and respiratory). Blood cultures, urine cultures and nasopharyngeal swabs were collected and empirical antibiotic treatment with Meropenem 500mg every 12 hours (Antibiotice S.A, Romania) and Linezolid 600mg every 12 hours (Infomed Fluids SRL, Romania) was initiated. Because the patient was recently discharged from the Urology Clinic where he received empirically these two antibiotics, we considered it appropriate to continue their administration until a pathogen was isolated. We note that the patient was admitted to the Urology Clinic for suspected urinary sepsis, which was ruled out by two negative urine cultures during that hospitalization.

**Table 1.** Laboratory tests and diuresis

**Laboratory test**	**Day 1**	**Day 3**	**Day 5**	**Day 7**	**Day 8**	**Day 9**	**Day 10**	**Day 12**	**Day 14**	**Day 16**	**Day 18**	**Day 20**	**Day 23**	**Reference Range**
Leukocytes	32500	28300	23700	20000	25100	27200	31100	27400	21600	17500	13100	9900	10000	3600–10000/μL
Neutrophils	28000	24200	20600	17300	21000	23700	26900	22100	16300	11400	9600	7800	7900	1400–6500/μL
Hemoglobin	9.33	9.4	9.2	9	8.9	8.8	8.8	9.1	9.4	9.85	9.6	9.3	9.1	13–17g/dL
Platelets	154000	143000	150000	149000	133000	121000	112000	117000	126000	123000	117000	119000	116000	150000–450000/μL
C reactive protein	140.5	122.5	110.5	70	140	219	243	194	152	93	40	10	15	0–5mg/L
Procalcitonin	14.54			5			12.3				8		6	<0.5mg/L
Presepsin	1369			714			1236				744		432	100–200 pg/mL
NT-proBNP	23614								21500					<125pg/mL
AST	24		25	18			22		18		23		30	5–34U/L
ALT	5		7	6			6		6		7		8	0–55U/L
Total bilirubin	1.42		1.37	1.27			1.22		1.09		1.26		1.41	0.2–1.2 mg/dL
Albumin	3.06						3.0							3.97–4.94 g/dL
Total protein	6.19						6.1							6.4–8.3 g/dL
INR	1.17		1.04		0.94			0.9		0.92			0.96	0.8–1.2
Sodium	136	134	134	133	133	132	132	131	130	130	130	130	130	136–145 mmol/L
Potassium	3.67	3.5	3.63	3.69	3.7	3.74	3.76	3.8	3.6	3.5	3.4	3.6	3.5	3.5–5.1 mmol/L
Glucose	94	106	101	95	88	78	83	96	99	94	121	117	104	70–105 mg/dL
pH	7.31	7.33	7.32	7.35	7.36	7.35	7.33	7.33	7.31	7.3	7.29	7.26	7.2	7.35–7.45
PO_2_	65	68	70	71	72	71	72	71	70	70	71	70	70	75–100 mmHg
PCO_2_	49	44	43	44	46	49	51	53	53	54	54	55	65	35–45 mmHg
P(A-a)O_2_	158,95	162,2	161,45	159,2	155,7	152,95	149,45	147,95	148,95	147,7	146,7	146,45	133,95	mmHg
FShunt	39	22	20	21	20	21	32	30	26	28	30	33	37	%
HCO_3_	18	19	21	22	23	22	20	19	19	18	18	17	20	22–26mEq/L
SaO_2_	75	88	90	89	90	89	84	85	87	86	85	80	78	95–100%
Lactate	3.6	3.5	3.4	3.1	3.2	3.3	3.5	3.1	2.9	2.8	3.0	3.1	3.2	<2mmol/L
Urea	117	89	87	81	70	74	72	71	72	79	85	82	79	19.04–44.08 mg/dL
Creatinine	2.85	2.31	2.23	2.01	1.62	1.85	1.9	1.76	1.79	2.07	2.11	2.15	2.16	0.72–1.25 mg/dL
Diuresis	3L	3.2L	3L	2.8L	2.9L	3.3L	3.1L	3L	2.7L	2.8L	2.6L	2.7L	2.5L	

**Fig. 1.**
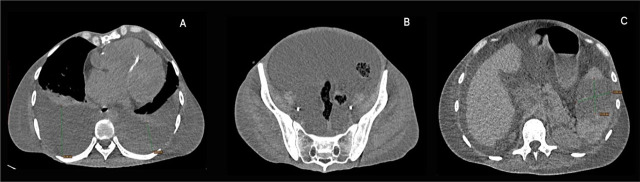
A. Chest CT scan: Large bilateral pleural effusions with atelectasis; Mild pectus excavatum with a Haller index of 2.28; B. Abdominopelvic CT scan: Large amount of ascites; C. Abdominal CT scan: Splenic infarcts

A subsequent non-contrast chest CT revealed large bilateral pleural effusions with partial lung atelectasis. In addition, a non-contrast CT scan of the abdomen and pelvis showed an enlarged liver without focal lesions, a large amount of ascites, and an enlarged spleen with multiple hypodense splenic lesions (suggestive of splenic infarction, which were also highlighted in the Urology Clinic through abdominal CT and abdominal ultrasonography) (Figure 1).

**Fig. 2.**
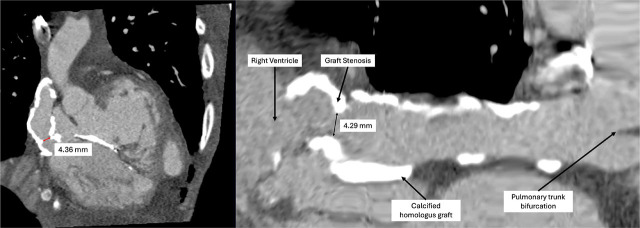
Cardiac CT scan with intravenous contrast: Homograft stenosis-reduction in the diameter of the conduit connecting the right ventricle to the pulmonary artery from 14 mm to 4.29 mm

**Fig. 3.**
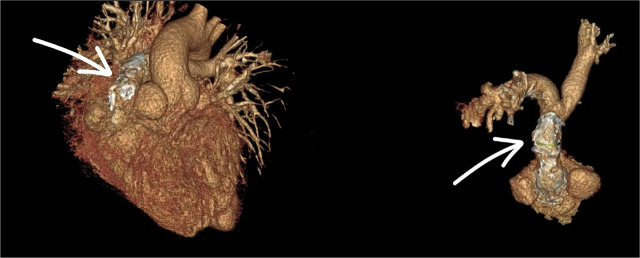
3D reconstruction of the heart showing the homograft conduit connecting the right ventricle to the pulmonary artery

A transthoracic echocardiography was performed in the emergency department and showed severe tricuspid regurgitation, severe pulmonary hypertension, and a decrease in left ventricular ejection fraction (LVEF) to 45% without any evidence of endocarditis (the last echocardiography, performed three months prior to admission, showed mild tricuspid regurgitation and a LVEF of 60%). A transesophageal echocardiography was attempted, but the patient was uncooperative and refused the procedure. Following these findings, we decided to admit the patient to the Internal Medicine Clinic for further investigation and specialised treatment. The patient’s condition remained stable, with a qSOFA score of 1 point, heart rate of 115/min, blood pressure of 110/70 mmHg without positive inotropes, oxygen saturation of 90% with a non-rebreather mask, and after intravenous hydration, serum creatinine decreased to 1.62 mg/dL (creatinine clearance 41 ml/min). Congestive heart failure treatment was initiated, including Ramipril 2.5 mg once a day (Helcor Pharma, Romania), Carvedilol 6.25 mg twice daily (Labormed, Romania), Spironolactone 25 mg once a day (Terapia, Romania), Dapagliflozin 10 mg once a day (AstraZeneca Pharma, Sweden) and Furosemide 100 mg a day by continuous intravenous infusion (Hameln Pharma GMBH, Germany). In addition, thromboprophylaxis with low-molecular-weight heparin, Enoxaparin 40mg (Sanofi, France), a proton pump inhibitor, Pantoprazole 40 mg (Rompharm Company, Romania) and an antipyretic, Paracetamol 1000mg (Braun, Germany) were administered. Two days after admission, the urine culture was positive for Candida lusitaniae and antifungal treatment with Fluconazole 200mg twice daily (Arena Group S.A, Romania) was initiated, while blood cultures and nasopharyngeal swabs remained negative. Given the improvement in renal function, we decided to perform a cardiac CT scan with intravenous contrast, which revealed a stenosis of the homograft connecting the right ventricle to the pulmonary artery (Figure 2, 3).

**Fig. 4.**
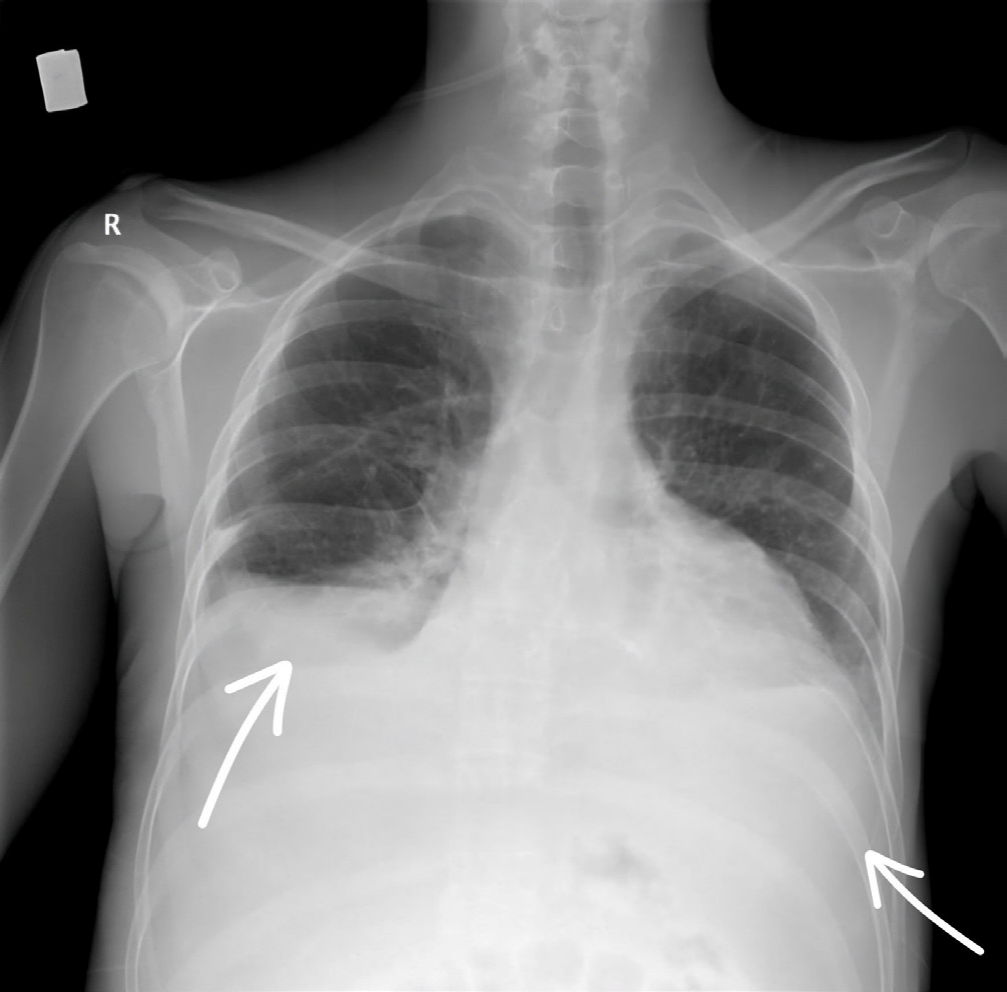
Chest X-ray after bilateral thoracentesis – no condensation foci, no signs of pneumothorax

After seven days of antibiotic treatment with Meropenem and Linezolid, the infectious syndrome partially subsided (leucocytes 20×10^3^/μL, neutrophils 17×10^3^/μL, C-Reactive Protein 70 mg/L, fever subsided). However, the patient’s clinical condition deteriorated, with marked dyspnea, tachypnea, oxygen saturation of 80% with non-rebreather mask, hemodynamic instability, heart rate of 125/min and blood pressure of 80/60 mmHg (qSOFA score of 2 points). A central jugular venous catheter was placed, and inotropic and vasopressor support with Noradrenaline 0,05 μg/kg/min up to 0,1 μg/kg/min (Amdipharm, Ireland) was initiated. Analyzing the cause of hypotension, we interpreted the drop in blood pressure as being more in the context of septic shock than cardiogenic shock. Given that septic shock is accompanied by peripheral vasodilation, we chose a positive inotropic agent with a vasoconstrictor effect. Furthermore, we considered that it was unlikely to be cardiogenic shock, as the patient’s echocardiography showed a left ventricular ejection fraction of 45%, which was sufficient to maintain cardiac output at rest. Thoracentesis and paracentesis were performed for therapeutic and diagnostic purposes, with evacuation of 1300 mL of clear serous pleural fluid and 4000 mL of ascites. Bacteriological tests were performed on both pleural and ascitic fluid, but no bacterial growth was detected. After the procedure, oxygen saturation increased to 95% with a non-rebreather mask, and the post-thoracentesis chest X-ray ruled out the presence of new congestive foci and pneumothorax (Figure 4). Given the evacuation of 4000 mL of ascites in a patient with albuminemia of 3g/dL and acute renal failure, we considered the administration of albumin to be inappropriate.

After ten days of antibiotic treatment with Meropenem and Linezolid, the patient became febrile again and complained of severe dyspnea, with an oxygen saturation of 84% on a non-rebreather mask and qSOFA score of 2 points; serial blood cultures were collected, but no bacterial growth was found. Additionally, we repeated the urine culture after administering the antifungal treatment, which did not reveal any bacterial or fungal growth. We consulted with infectious disease specialists and interpreted the sepsis as infective endocarditis according to the Duke criteria and stopped the antibiotic treatment with Meropenem and Linezolid and started Vancomycin 500mg every 12 hours (Rompharm, Romania) and Gentamicin 80mg once a day (KRKA, Slovenia) instead. After changing the antibiotic regimen, the fever subsided, leucocytes normalised to 9.9×10^3^/μL, neutrophils to 7.8×10^3^/μL, C Reactive Protein to10 mg/L, and the patient’s clinical condition improved, with a blood pressure of 110/60 mmHg without inotropic support and an oxygen saturation of 96% with a non-rebreather mask. As the patient’s kidney function improved, antibiotic doses were adjusted as follows: Vancomycin 1 g every 12 hours and Gentamicin 80 mg every 12 hours. After 13 days of antibiotic treatment, the patient exhibited marked dyspnea with an oxygen saturation of 78% on a non-rebreather mask. Arterial blood gas analyses indicated the presence of hypercapnic respiratory acidosis, which prompted the decision to transfer the patient to the intensive care unit for airway management. Subsequently, the patient experienced cardiorespiratory arrest due to asystole. The Basic Life Support (BLS) and Advanced Life Support (ALS) protocols were initiated, and the patient was orotracheally intubated and mechanically ventilated. In addition, 1 mg of Adrenaline was administered every 4 minutes. However, despite these measures, the patient remained unresponsive, and death was subsequently declared.

## Discussion

The Rastelli procedure was first described in literature by Giancarlo Rastelli in 1967 and the first successful surgery through this technique underwent at the Mayo Clinic in 1968. Being a complex open-heart surgical procedure, it has brought substantial benefits both for the correction of great arteries transposition and the burden of long-term complications. Studies show high survival rates immediately after the procedure, with some reporting rates as high as 94%. Although early survival rates are favorable, long-term survival rates vary. Some studies report 10-year survival rates of around 80%, and around 50% of patients survive for up to 20 years. Conduit replacement is necessary for most patients, regardless of the repair technique, but it can now be performed with reduced morbidity and mortality [5]. With a focus on strategies to mitigate the necessity for subsequent interventions, a study conducted at the German Heart Center Munich determined that patients aged 4 or older at the time of the Rastelli procedure exhibit a reduced requirement for surgical reinterventions for conduit replacement. Nonetheless, the study strongly recommended early Rastelli repair, acknowledging that patients in this age group demonstrate an elevated risk of long-term mortality [9]. In addition to conduit stenosis (the most well-known late complication of this procedure), several others have been described. These include obstruction of the left ventricular outflow tract, biventricular dysfunction, late arrhythmias, and infectious endocarditis [10]. Our patient experienced two of these complications, the management of which we will detail below.

When faced with a hyperpirexic patient with signs and symptoms of heart failure, the first imaging strategy was transthoracic echocardiography performed in the emergency department, revealing the first changes that could relate to the patient’s symptoms, namely newly developed, severe tricuspid regurgitation and a decrease in left ventricular ejection fraction to 45%. However, given the patient’s history of the Rastelli procedure, we considered the possibility that the severe tricuspid regurgitation discovered on echocardiography was secondary to pressure and volume overload of the right ventricle caused by conduit stenosis, this being the most common complication and cause of reintervention, as stated before [11]. To assess the integrity of the homograft, we performed a cardiac computed tomography study augmented by intravenous contrast, which confirmed the suspicion of graft stenosis. Although the cardiac CT study aided in diagnosing this complication, it is important to note that magnetic resonance imaging would have allowed a better quantification of the integrity of the homograft, especially in adults after surgery [12, 13], but this could not be performed due to technical unavailability. In order to determine the cause of hyperpirexia, in addition to bacteriological cultures, we considered performing a contrast-enhanced computed tomography study of the abdomen and pelvis, which ruled out any additional or alternative source of infection, while revealing splenic infarctions. This imaging find led to raising the suspicion of infectious endocarditis.

The main causes of splenic infarction can be grouped into several categories. Embolic causes are related to infectious endocarditis, valve prostheses or cardiac prosthetic materials, atrial fibrillation (excluded by EKG beforehand), recent myocardial infarction (excluded by EKG and echocardiography), atrial myxoma (excluded by echocardiography). Thrombotic causes include severe atherosclerosis (excluded by normal lipid profile, normal calcium score on thoracoabdominal CT), splenic artery thrombosis, dissected aortic aneurysm involving the origin of the splenic artery (both excluded by thoracoabdominal CT), systemic vasculitis (such as systemic lupus erythematosus; phenotype and symptoms did not concur with vasculitis). Hematological causes include sickle cell anemia, myeloproliferative neoplasms, which were ruled out through laboratory tests such as complete blood count, normal peripheral blood smear. Infectious causes (e.g. mononucleosis or malaria) and traumatic causes were excluded based on medical history and physical examination.

Although the transthoracic echocardiography showed no evidence of vegetations, and blood cultures were negative, the patient refused the transesophageal echocardiography. Facing this scenario, the modified Duke criteria [14] supported the diagnosis of infective endocarditis by fulfilling one major criterion: new-onset severe tricuspid regurgitation, and three minor criteria: predisposing cardiac conditions (surgically corrected congenital heart defect, presence of the Gore-Tex patch sealing the ventricular septal defect and the homograft connecting the right ventricle to the pulmonary artery), fever and vascular phenomena (splenic infarcts). A similar case was described in literature in an immunocompromised patient with Rastelli conduit infection, in whom no signs of infective endocarditis could be detected by echocardiography, but which was revealed by a fludeoxyglucose positron emission tomography scanning (imaging investigation not available in our hospital) [15].

Administering antibiotic treatment to patients with impaired renal function is challenging. Many antibiotics require dose adjustment based on creatinine clearance, due to renal excretion. In this patient’s case, three out of four antibiotics required adjustment: Meropenem, Vancomycin and Gentamicin. When choosing drug classes, we initially opted for broad-spectrum antibiotic therapy because the patient’s condition was critical at admission and the inflammatory markers were significantly elevated. Furthermore, as this was a patient who had been hospitalized multiple times for sepsis of unknown origin and had recently been discharged from the urology department (receiving treatment with Meropenem and Linezolid), we decided to continue this regimen as the patient had most likely developed resistance to narrow-spectrum antibiotics following repeated courses of antibiotics. Due to the ineffectiveness of the Meropenem and Linezolid combination, we consulted with infectious disease specialists, who recommended empirical treatment with Vancomycin and Gentamicin, these being the preferred antibiotics for endocarditis with negative blood cultures, according to the latest European Society of Cardiology guidelines on infectious endocarditis, published in 2023 [16]. When comparing this case with other references researched in literature, we found reports of endocarditis cases with negative blood cultures that were later found to be of mycotic etiology [17]. The latest update on therapeutic management of fungal infective endocarditis recommends using Amphotericin B alone or with Flucytosine or high doses of Echinocandin, followed by de-escalation to Fluconazole or Voriconazole [18]. However, given the absence of first-line antimycotics at our disposal, this treatment could not have been initiated. However, our patient had been receiving antimycotic treatment with Fluconazole from the beginning of hospitalization for a urinary tract infection with Candida lusitaniae.

The etiology and pathophysiological mechanism of polyserositis is also an important issue to be discussed. Polyserositis is characterized by fluid accumulation in several serous cavities of the body: pleurisy, pericarditis, ascites. In our patient’s case, we interpreted polyserositis as secondary to right heart failure. In this context, the main pathophysiological mechanism is the right ventricles’ inability to pump blood into the pulmonary circulation, resulting in systemic venous stasis. Thus, central venous pressure will increase and, implicitly, hydrostatic pressure in the systemic capillaries will also increase, leading to fluid accumulation from capillaries into serous spaces (pleural, pericardial, peritoneal). Furthermore, hypoproteinemia can cause, or contribute to, polyserositis by decreasing colloid-osmotic pressure in the capillaries.

As for the cause of death, considering the patient’s past struggle with chronic respiratory failure (exhibiting oxygen saturation levels of around 80–85% in ambient air), the abrupt clinical and paraclinical decline triggered by the onset of severe dispnoea, tachypnea and hypercapnic respiratory acidosis led to a further exacerbation of chronic respiratory failure. Thus, considering the acute on chronic respiratory failure, we concluded that stenosis of the conduit was the main cause of death. The conduit stenosis mechanism was due to a combination of compression caused by pectus excavatum and further degenerative processes, as evidenced by extensive calcifications on cardiac CT. Given the chronic nature of the conduit’s degenerative process and the sudden worsening of the clinical picture, we establised that the infectious endocarditis on a foreign body to be the mechanism for the worsening of stenosis. Additionally, we interpreted the newly developed tricuspid regurgitation as an adaptive response to volume overload and increased right ventricular pressure. Given the splenic infarctions present in our patient, we can reasonably accept the presence of an embolic source, in the context of endocarditis, releasing septic emboli into the bloodstream that reach cerebral and pulmonary circulations. Due to the rapid progression of events and the patient’s critical condition, a cranial CT scan could not be performed to confirm or rule out an ischemic stroke. Furthermore, we hold no evidence to sustain the release of septic emboli into the pulmonary circulation, which could have led to acute respiratory failure. A relevant case in literature is that of a 19-year-old male, diagnosed with infectious endocarditis with Candida spp, complicated by pulmonary infarction and pulmonary mycotic abscesses, who died of massive pulmonary hemorrhage, diagnosed postmortem, 6 months after undergoing a second Rastelli operation for conduit stenosis [19]. Another factor considered was septic shock in the context where antibiotic treatment was not adequate without clear pathogen isolation. However, due to the decrease in inflammatory markers, we are able to sustain that sepsis was not the decisive factor that caused death. Definitive autopsy findings are unavailable to us, as the patients’ family refused the procedure (as permited by current law procedures in our country).

## Conclusions

Managing a patient with a surgically corrected congenital heart malformation can be a real challenge. In the case of a patient with great arteries transposition that has been corrected by the Rastelli procedure, it is always necessary to consider the potential late complications that may arise. Since the procedure involves the implantation of a foreign body, the risk of infection is increased and must be evaluated every time the patient is symptomatic. Though vegetation may elude detection through imaging and pathogen isolation can fail if blood dissemination is absent, evaluation by Duke criteria can establish the diagnosis of infective endocarditis. In our case, splenic infarctions were the key to the diagnosis. On the other hand, the presence of signs of right-sided heart failure and new-onset severe tricuspid regurgitation always raise the suspicion of homograft stenosis, which must be investigated by imaging with cardiac CT scan with intravenous contrast or cardiac MRI.
